# Effect of Selective Androgen Receptor Modulator Enobosarm on Bone Healing in a Rat Model for Aged Male Osteoporosis

**DOI:** 10.1007/s00223-020-00751-x

**Published:** 2020-09-02

**Authors:** Marina Komrakova, Janek Nagel, Daniel Bernd Hoffmann, Wolfgang Lehmann, Arndt Friedrich Schilling, Stephan Sehmisch

**Affiliations:** Department of Trauma Surgery, Orthopaedics and Plastic Surgery, University Medical Center Goettingen, Robert-Koch Str. 40, 37075 Goettingen, Germany

**Keywords:** Selective androgen receptor modulator, Enobosarm, Testosterone, Bone healing, Male osteoporosis

## Abstract

Enobosarm (ostarine, MK-2866, or GTx-024) is a non-steroidal selective androgen receptor modulator. This study evaluated the effect of various regimens of enobosarm (EN) on bone healing in an orchiectomized rat model for aged male osteoporosis and compared it to testosterone (T) treatment. Ninety eight-month-old male Sprague Dawley rats were either orchiectomized (Orx) or left intact (Non-Orx) and divided into groups (*n* = 15/group): (1) Non-Orx; (2) Orx; (3) Orx+T-th; (4) Orx+EN-th; (5) Orx+T-pr; and (6) Orx+EN-pr. Prophylaxis (Pr) treatments were applied immediately after Orx for up to 18 weeks. Therapy (Th) treatments were applied 12 weeks after Orx for up to 6 weeks. Bilateral tibia osteotomy with plate osteosynthesis was performed 12 weeks after Orx in all groups. EN and T were mixed with the diet; the daily dosage was 0.35 ± 0.06 and 41 ± 8 mg/kg BW, respectively. Both T treatments improved bone healing by increasing callus volume and area, bone volume and density, and cortical width; they had no effect on prostate or levator ani weight. EN-pr increased the callus area and callus density and decreased cortical density, but increased prostate weight. The effect of T-pr and T-th on bone was stronger than EN-pr. EN-th affected bone healing negatively by reducing callus density and area and delaying osteotomy bridging. Levator ani weight was increased in both EN groups. EN treatment after fracture is not advisable in aged males. EN-pr treatment as a therapy for bone healing in men could be further investigated; endocrinological side effects must be closely monitored.

**Electronic supplementary material:**

The online version of this article (10.1007/s00223-020-00751-x) contains supplementary material, which is available to authorized users.

## Introduction

Fractured bones impair the quality of life of patients, and osteoporosis impedes normal bone healing processes [1, 2]. Despite increasing recognition of the problem of male osteoporosis, postmenopausal osteoporosis has been the focus of research, and most treatments have been developed for women. In men, there are only a few options for osteoporosis treatment. Non-pharmacologic treatment includes diet and lifestyle changes to reduce the risk of fracture [3, 4]. Therapeutic options include the administration of bisphosphonates, human parathyroid hormone, teriparatide, denosumab, or romosozumab. These treatments are prescribed to prevent osteoporotic fractures. There is, however, no approved treatment for support of a fast and undisturbed healing process [2].

Testosterone is an essential hormone for the maintenance of bone and muscle mass in men. In males, osteoporosis occurs primarily due to diminished testicular testosterone production. Testosterone supplementation offers a treatment for this condition [5, 6]. The major limitations of testosterone replacement therapy in elderly men are negative side effects, so its benefits should be weighed against the risks of replacement [6, 7].

Recently, the application of non-steroidal selective androgen receptor modulators (SARMs) has been suggested for the treatment of osteoporosis and frailty [8, 9]. Enobosarm (ostarine, MK-2866, or GTx-024) is a non-steroidal SARM that binds to the androgen receptor (AR) with tissue selectivity and cannot be converted to dihydrotestosterone and estrogen [10]. Therefore, it is thought to cause fewer side effects than testosterone [9, 11]. Treatment with enobosarm is not approved for human use in any country so far; however, it is sold on the internet [10]. It has been used by athletes and was banned by the World Anti-Doping Agency (https://www.wada-ama.org/en/content/what-is-prohibited/prohibited-at-all-times/anabolic-agents, retrieved 19 May 2020). Enobosarm has been studied in several phase-II and phase-III clinical trials in patients with cancer cachexia, sarcopenia, breast cancer, and stress urinary incontinence [12–15]. It reportedly improves muscle mass, showing a beneficial effect on bone in experimental studies [9, 16, 17]. Recently, we showed a favorable effect of enobosarm treatment on bone healing in a rat model of postmenopausal osteoporosis [18]. This raises the question of whether these results can be extended to bone healing in male rats. Gender differences in response to SARMs have not been studied as of yet, and most reports present either combined data for both sexes or single-sex data [9, 12, 13, 17, 18].

The present study evaluated the effect of various regimens of enobosarm on bone healing in an orchiectomized rat model for aged male osteoporosis and compared it to treatment with testosterone.

## Materials and Methods

### General Procedures

**Fig. 1:**
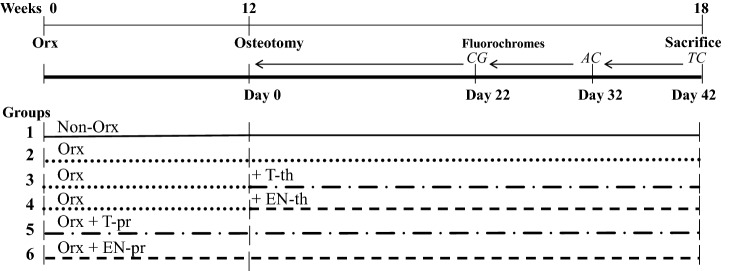
Schematic flowchart of the experiment. Eight-month-old male rats were either orchiectomized (Orx) or left intact (Non-Orx). Bilateral tibia osteotomy was performed 12 weeks after Orx in all groups. Treatments with testosterone (T) or enobosarm (EN) were started either immediately after Orx (pr) or after osteotomy (th). Fluorochromes were s.c.-injected: calcein green (CG) on day 22, alizarin complexone (AC) on day 32, and tetracycline hydrochloride (TC) on day 42 after osteotomy. Samples were collected 6 weeks after osteotomy

Ninety eight-month-old male Sprague Dawley® rats (Janvier, Le Genest-Saint-Isle, France) were used in the experiment (Fig. 1). Seventy-five rats were bilaterally orchiectomized (Orx). Fifteen rats were left non-orchiectomized (Non-Orx) to serve as a healthy control group (group 1). Anesthesia was as follows: intraperitoneal (IP) injection of ketamine (Inresa Arzneimittel GmbH, Freiburg, Germany), xylazine (Xylariem, Pharma-Partner Vertriebs GmbH, Oostkamp, Belgium), and midazolam (Rotexmedica GmbH, Trittau, Germany) (80 mg, 4 mg and 2.5 mg/kg body weight [BW], respectively). After eight rats died, the anesthesia was changed to isoflurane inhalation with the addition of subcutaneous (SC) injection of carprofen (5 mg/kg BW, Caprive, Bayer Vitalo GmbH, Leverkusen, Germany) and no further losses were observed.

The Orx rats were divided into five groups (groups 2–6, 15 rats each). In group 2, the Orx rats were left untreated to serve as osteoporotic controls (Fig. 1). In groups 3 and 4 (Orx+T-th, Orx+EN-th), treatments with testosterone propionate (T) or enobosarm (EN), respectively, were started 12 weeks after Orx and were applied for up to 6 weeks (therapy [th]). In groups 5 and 6 (Orx+T-pr, Orx+EN-pr), rats were treated either with T or with EN, respectively, immediately following Orx for up to 18 weeks (prophylaxis [pr]).

EN and T were mixed with a soy-free diet by Ssniff Special Diets GmbH (Soest, Germany) at a concentration of 8.56 mg and 1 g per kg of diet, respectively, to achieve a daily EN dosage of 0.4 mg/kg body weight (BW) [17, 18], and a T dosage of 50 mg/kg BW [19–21]. EN (Purity > 98%, No.:841205-47-8) was obtained from Shanghai Biochempartner Co. Ltd. (Shanghai, China) through the distributor Hölzel Diagnostika (Cologne, Germany). Testosterone propionate was supplied by Sigma-Aldrich (St. Louis, USA). All rats were fed with a soy-free pelleted diet (Ssniff Special Diets GmbH) [22] and had free access to food and demineralized water throughout the experiment. The food intake was calculated by a weekly weighing of the remaining food in the cage. The daily dosage calculated at the end of the experiment averaged over the treatment weeks to 0.35 ± 0.06 mg/kg BW for enobosarm and 41 ± 8 mg/kg BW for testosterone (Table 1).

**Table 1:** Food intake, BW, doses of T and EN averaged over the weeks and other parameters, measured at the end of the study in Non-Orx and Orx rats treated with enobosarm (EN) or testosterone propionate (T) applied either after Orx (pr, weeks 0–18) or after osteotomy (th, weeks 12–18)

Parameters	Non-Orx		Orx		Orx+T-th		Orx+EN-th		Orx+T-pr		Orx+EN-pr	
	Mean	SD	Mean	SD	Mean	SD	Mean	SD	Mean	SD	Mean	SD
Mean food intake (g/rat/day)	29	5	28	6	30	5	29	4	28	6	29	6
Mean BW (g)	712	36	654^a^	41	666^a^	31	650^a^	31	649^a^	34	664^a^	37
Mean T or EN dose (mg/kg BW/day)					36	9	0.30	0.02	43	8	0.36	0.05
*Serum*												
OC (ng/mL)	175	24	208	54	200	33	123^bd^	27	183	32	139^bd^	26
ALP (U/L)	175	57	134	38	147	35	194	43	151	28	173	71
CTX-I (ng/mL)	17	3	18	4	19	3	17	1	20	2	17	4
Magnesium (mmol/L)	0.74	0.10	0.68	0.07	0.75	0.08	0.70	0.05	0.77	0.06	0.74	0.07
Calcium (mmol/L)	2.17	0.20	2.06	0.16	2.21	0.22	2.03	0.11	2.28	0.12	2.16	0.14
Phosphor (mmol/L)	1.84	0.26	1.56	0.19	1.69	0.24	1.79	0.22	1.99^b^	0.25	2.03^b^	0.27
*Weights*												
BW (g)	727	78	642	69	678	122	629	73	659	80	667	105
Levator ani (g)	0.64	0.25	0.31^a^	0.08	0.32^a^	0.05	0.57^b^	0.08	0.37^a^	0.11	0.54^b^	0.12
Prostata (g)	1.22*	0.30	0.18	0.07	0.19	0.07	0.39	0.08	0.34	0.13	0.62^bcd^	0.13
M. gastrocnemius (g)	3.2	0.5	2.8	0.5	3.0	0.6	3.1	0.4	2.9	0.6	2.9	0.6
M. soleus (g)	0.29	0.05	0.27	0.05	0.27	0.03	0.30	0.04	0.26	0.07	0.26	0.06
*Biomechanics*												
Stiffness (N/mm)	75	29	75	32	82	41	66	35	64	28	82	41
Yield load (*N*)	56	30	44	14	63	17	79	44	57	24	73	42
*Microradiography*												
Dorsal												
Ct.Wi.d (mm)	0.58	0.11	0.49	0.17	0.67^b^	0.18	0.53	0.08	0.64^b^	0.21	0.52	0.10
Ct.Dn.d (%)	99.4	0.5	99.0	1.1	99.5	0.8	98.8	0.8	99.3	0.7	99.2	0.5
Cl.Wi.d (mm)	0.92	0.41	0.70	0.32	0.89	0.48	0.99	0.58	1.1	0.50	0.80	0.43
Cl.Dn.d (%)	67.6	17.5	61.6	16.0	60.2	12.9	52.7^a^	13.8	58.2	11.9	65.0	18.0
Endosteal												
Cl.Dn.e (%)	88.7	3.9	76.1	10.8	69.6^a^	15.1	67.4^a^	13.9	70.5^a^	14.3	69.9^a^	15.9
*Fluorescence analysis*												
The time of the first bridging (day)	27	7	26	9	26	6	34	8	29	9	27	8

*Differs from all other groups

^a^Differs from Non-Orx

^b^Differs from Orx

^c^Differs from Orx+T-pr

^d^Differs from Orx+T-th (Tukey test, *p* < 0.05)

After 12 weeks post-orchiectomy, all rats underwent a bilateral metaphyseal osteotomy of the tibia [23]. Briefly, a transverse osteotomy (0.5 mm) was created using an ultrasound saw (Piezosurgery®, Mectron Medical Technology, Carasco, Italy). The tibia was fixed with the aid of a T-titan plate and four screws (Stryker Trauma, Selzach, Switzerland). During the operation, the rats were anesthetized by IP 75 mg ketamine, 5 mg midazolam per kg BW, SC 0.1 mg/kg BW buprenorphine (Temgesic® RB Pharmaceuticals Limited, Berkshire, UK), and isoflurane inhalation. After ten rats died, the dosage of ketamine and midazolam was reduced (38 mg and 2.5 mg/kg BW, respectively) and no further losses were observed.

Postoperative pain therapy was given with buprenorphine (SC) and metamizol (drinking water). Buprenorphine was applied as follows: days 1–2 post-osteotomy, 0.1 mg/kg BW 3 times/day; day 3, 0.1 mg/kg BW twice/day, days 4–5, 0.05 mg/kg BW twice/day; days 6–10, 0.05 mg/kg BW once/day. Metamizol was dissolved in demineralized water (20 g/L, Novaminsulfon, Winthrop Arzneimittel GmbH, Frankfurt am Main, Germany) After 10 days, the complications (dehydration, obstipation, ileus, and death [*n* = 15]) were observed and the pain therapy was immediately interrupted. During week 14, food intake was not measured due to complications from the postoperative pain therapy. During this week, the rats were fed additionally with oat flakes and bread, and received 1–2 drops/day of Herbi Colan (WDT, Garbsen, Germany) for regulation of digestive processes.

During the further healing period, the newly built callus was labeled by SC injection of fluorescent dyes [22]. Calcein green (CG, 10 mg/kg BW, Waldeck GmbH, Münster, Germany) was applied on day 22, alizarin complexone (AC, 30 mg/kg BW, Merck KGaA, Darmstadt, Germany) on day 32, and tetracycline hydrochloride (TC, 25 mg/kg BW, Carl Roth GmbH+Co. KG, Karlsruhe, Germany) on day 42 after osteotomy, respectively (Fig. 1).

Animals were decapitated under CO_2_ anesthesia 6 weeks after osteotomy. Blood samples were collected for further analysis. Heart, liver, kidney, spleen, *musculus gastrocnemius* (M. gastrocnemius), M. soleus, M. levator ani, and prostata were extracted and weighed. After removal of soft tissues and extraction of the plates and screws, both tibiae were stored at − 20 °C for further micro-CT, biomechanical, and histological analysis. At the end of the experiment, the following number of animals could be analyzed in each group: Non-Orx, 12; Orx, 8; Orx+T-th, 5; Orx+EN-th, 7, Orx+T-pr, 9, and Orx+EN-pr, 9.

### Serum Analysis

Alkaline phosphatase (ALP), magnesium, calcium, and phosphor were measured in serum at the Department of Clinical Chemistry, University Medical Center, Goettingen, Germany. Analyses were performed using an automated chemistry analyzer (Architect c16000 analyzer, Abbott, Wiesbaden, Germany) and commercially available kits (Architect, Abbott) according to the manufacturer’s instructions. Osteocalcin (OC) and Cross Linked C-telopeptide of Type I Collagen (CTX-I) were assessed using commercial analysis kits, rat-MID™ Osteocalcin EIA and RatLaps (CTX-I) EIA (Immunodiagnostic Systems GmbH, Frankfurt am Main, Germany).

### Analysis of Bone Healing

#### Micro-computed Tomographical (micro-CT) Analysis

Both tibiae were scanned using a Quantum FX micro-CT (Caliper Sciences, Hopkinton, MA, USA). The scan protocol was as follows: 70 kVp, 200 μA, 2-min exposure time, 360° rotation, 3600 projections, 20 × 20 mm^2^ field of view, 512-pixel matrix, and 40 × 40 × 40 μm^3^ effective voxel size [18, 23]. A phantom block with five hydroxyapatite elements of several mineral densities was scanned with each tibia to convert the data into bone mineral density (g/cm^3^). The scans were analyzed with the program developed in our laboratory [18, 23]. The measurement area extended 1.5 mm proximally and distally from the osteotomy line. The parameters assessed were as follows: total bone mineral density (total BMD), bone mineral density and bone volume (bone BMD and bone volume), osseous callus BMD and volume (callus BMD and callus volume), and osseous callus fraction (osseous callus V/total callus V) [23].

#### Biomechanical Analysis

A three-point bending test was applied using a testing device (Zwick/Roell, type 145660 Z020/TND, Ulm, Germany), as previously described in detail [24]. The roller stamp was loaded at the osteotomy line at the tibial tuberosity at a feed motion rate of 5 mm/min. The test was stopped automatically by the software (testXpert, Zwick/Roell) before plastic deformation ended with a bone fracture. Stiffness (N/mm), the slope of the linear rise of the curve during elastic deformation and yield load (*N*), end point of elastic deformation were calculated using MS Excel (MS Office 2016) [24].

#### Histological Analysis

**Fig. 2:**
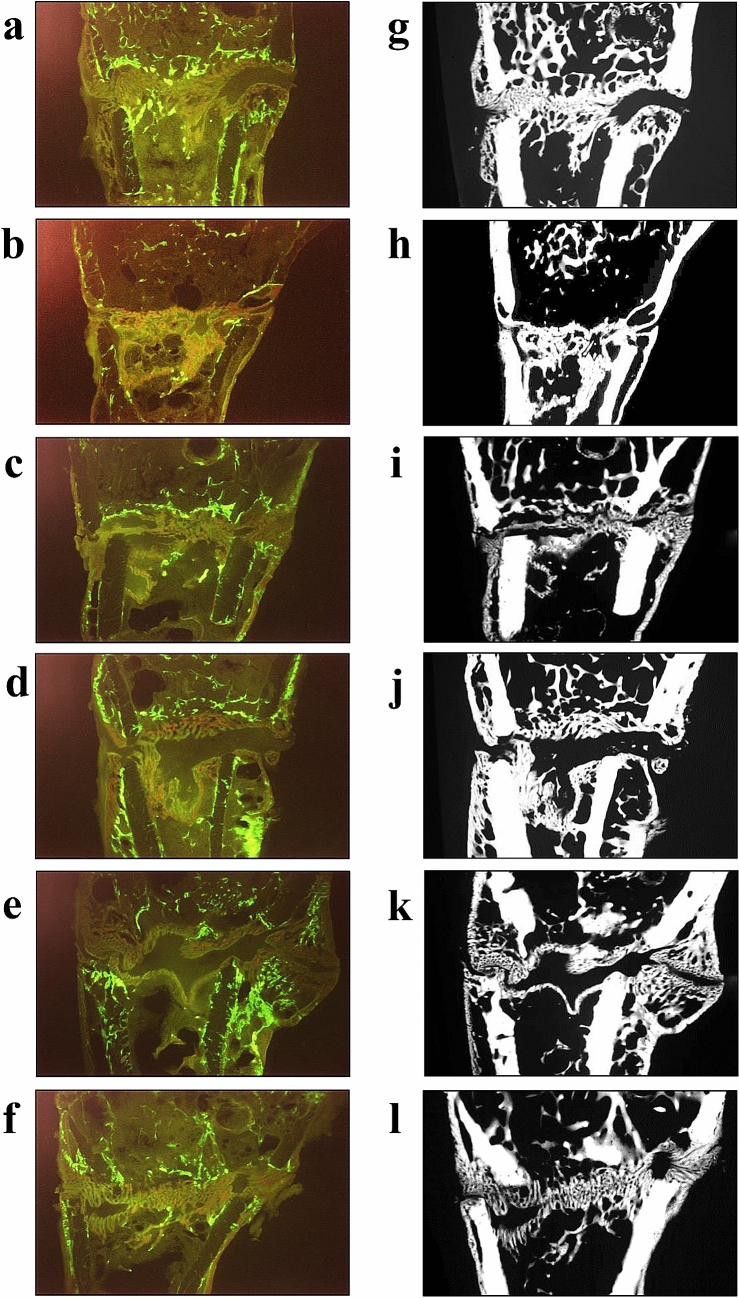
Longitudinal sections of the tibia metaphysis at osteotomy site labeled with fluorochromes (**a**–**f**) and corresponding microradiographs (**g**–**l**) made 6 weeks after osteotomy in the treatment groups: (**a**, **g**) Non-Orx (**b**, **h**), Orx (**c**, **i**), Orx+T-th (**d**, **j**), Orx+EN-th (**e**, **k**), Orx+T-pr (**f**, **l**), and Orx+EN-pr

Tibiae were embedded in methyl methacrylate (Merck, Darmstadt, Germany), and 150-µm-thick longitudinal sections of tibiae were cut using a diamond saw microtome (Leica SP1600, Leica Instruments GmbH, Nussloch, Germany) [23, 24]. The time of the first osseous bridging of the osteotomized bone ends was determined visually using a microscope (Leica, Leitz DM RXE) by analyzing all sections (at least ten). The CG-stained callus was built within 0–22 days after osteotomy, the AC-stained callus was formed within 23–32 days, and the TC-stained callus tissue was built within 33–42 days after osteotomy [22]. Three central representative histological sections (Fig. 2a–f) were chosen to measure the total area (Tt.Ar.) and labeling-specific area (CG, AC, and TC) of the callus. These sections were microradiographed using a Faxitron Cabinet X-ray system (Hewlett–Packard, Buffalo Grove, IL, USA) and Kodak Industrex film (SR45, 100 NIF, Kodak, Paris, France). Using microradiographs (Fig. 2g–l), cortical width and density distal to osteotomy (Ct. Wi. and Ct. Dn., respectively), periosteal, and endosteal callus width and density (Cl. Wi. and Cl. Dn., respectively) were determined [23]. The measurement area of the tibia was 1.5 mm proximally and distally from the osteotomy line. Three regions were identified: ventral, plate side (v); dorsal, opposite site (d); and (e) endosteal part [23, 24].

### Statistical Analysis

Statistical analyses were performed using GraphPad Prism (Version 5.04, GraphPad Software, Inc., San Diego, CA). A one-way analysis of variance (ANOVA) and the Tukey test were applied (*p* < 0.05) to analyze differences between the treatment groups. Data are shown as means and standard deviations (SD).

## Results

### Animal Model

BW and food intake did not differ significantly between the groups if analyzed weekly (Suppl. Figure 1). BW averaged 702 g (from 542 to 830 g, SD = 75 g) at the beginning of the study and 673 g (506–870, SD = 88 g) at the end of the study. Analysis of BW over the weeks showed significantly lower BW in all five Orx groups compared with the Non-Orx group (Table 1). Food intake over the weeks was not different among the groups (Table 1). Daily food intake of rats was 29 ± 5 g on average. Food intake as well as BW decreased during the 1st week after the osteotomy operation (week 13, Suppl. Figure 1).

Dosage of EN and T did not differ between the th and the pr groups (Table 1). In both groups, the dosage decreased in correspondence with the decreased food intake after osteotomy (Suppl. Figure 1).

The weight of the heart, liver, kidney, and spleen did not differ among the treatment groups (data not shown). The weight of M. levator ani was higher in the Non-Orx group compared with the Orx, Orx+T-th, and Orx+T-pr groups (Table 1). Both EN treatments (th and pr) increased the weight of M. levator ani compared to the Orx rats. Prostate weight was significantly higher in Non-Orx rats than in the Orx groups (Table 1). In the Orx+EN-pr group, the weight of the prostate was higher than in the Orx, Orx+T-th, and Orx+T-pr groups. The weight of M. gastrocnemius and M. soleus did not differ significantly between the groups (Table 1).

### Serum Analysis

Serum levels of ALP, CTX-I, magnesium, and calcium did not differ among the groups, whereas phosphorus was elevated in the Orx+T-pr and Orx+EN-pr groups compared with the Orx group (Table 1). The OC level was decreased in both the EN-treated groups compared with the Orx and Orx+T-th groups (Table 1).

### Bone Healing Analysis

**Fig. 3:**
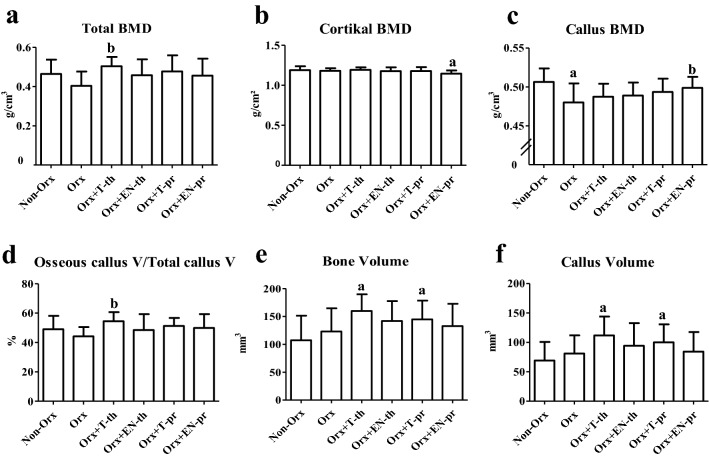
Micro-CT analysis performed at the tibia metaphysis 6 weeks after osteotomy. **a** Total BMD. **b** Cortical BMD. **c** Osseous callus BMD. **d** Osseous callus volume/total callus volume. **e** Bone volume. **f** Osseous callus volume. Mean ± standard deviation. **a** Differs from Non-Orx. **b** Differs from Orx (*p* < 0.05)

Micro-CT analysis at the osteotomy site revealed increased total BMD and osseous callus fraction in the Orx+T-th group compared with the Orx group (Fig. 3a, d). Callus BMD was significantly lower in the Orx group than in the Non-Orx group. In the Orx+EN-pr group, callus BMD was higher than in the Orx group, whereas cortical BMD was reduced compared with that of the Non-Orx group (Fig. 3b, c). Both T treatments (th and PR) elevated bone and callus volume (Fig. 3e, f). Bone BMD and cortical volume were not different among the groups (data not shown).

Biomechanical analysis did not show any significant differences in yield load or stiffness of the tibiae among the treatment groups (Table 1).

Analysis of histological sections of tibia revealed delayed osseous bridging in the Orx-EN-th group, whereas all other Orx groups and the Non-Orx rats showed comparable occurrences of the first osseous bridging (Table 1).

**Fig. 4:**
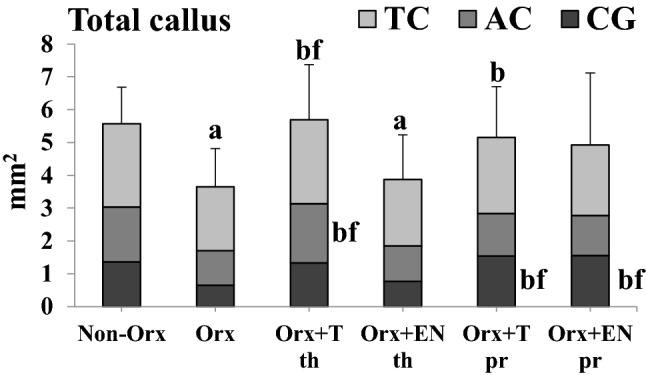
Total callus areas (mm^2^) measured according to the fluorescence-labeled areas (calcein green [CG], alizarin complexone [AC], and tetracycline [TC]). Mean ± standard deviation. (a) Differs from Non-Orx. (b) Differs from Orx. (f) Differs from Orx+En-th (*p* < 0.05)

Quantitative analysis of fluorescence-stained callus showed that more callus was built in the Orx+T-pr and Orx+EN-pr groups compared with the Orx group during the first 3 weeks of healing (CG staining), whereas in the Orx group, total callus formation was delayed (Fig. 4). During weeks 4 and 5 of healing (AC stainings), T-th treatment caused increased callus formation, whereas within the final weeks of healing (TC staining), no differences were recorded between the groups (Fig. 4). The amount of total callus was lower in the Orx and Orx+EN-th groups than in the Non-Orx group and higher in the Orx+T-th and Orx+T-pr groups compared with the Orx group (Fig. 4).

Analysis of microradiographs showed thicker cortical bone in the Orx+T-th and Orx+T-pr groups than in the Orx group and lower callus density in the Orx+EN-th group compared with the Non-Orx group at the dorsal aspect (Table 1). Endosteal callus density was lower in all T- and EN-treated groups than in the Non-Orx rats (Table 1). At the ventral aspect, no significant differences were observed between the groups (data not shown).

## Discussion

Our results showed that T treatments elicited a stronger effect on bone healing than EN treatments in the orchiectomized rat model. Both testosterone treatments (T-pr and T-th) were effective in improving bone parameters such as callus volume and area, bone volume and density, and cortical width, irrespective of duration of treatment. Similar to the T-pr treatment, the EN-pr treatment increased callus area formed during the 1st weeks of healing, and additionally enhanced callus density and decreased cortical density. The EN-th treatment affected bone healing negatively, reducing callus density and area and delaying the occurrence of osseous bridging.

Orx impaired callus formation during the early stages of bone healing, and this effect remained until the end of the study. The orchiectomized rat model applied in the present study is often used to study male osteoporosis and osteoporotic bone healing [22, 25–27]. Orchiectomy was confirmed by the absence of testis and by the reduction in prostata and levator ani weights [28].

BW, food intake, weight of organs, and leg muscles were not affected by either Orx or EN and T treatments. The analysis of BW over experimental weeks revealed a lower BW in all Orx rats than in Non-Orx rats. This decrease in BW as a consequence of the Orx is a known phenomenon in rats that occurs independently of food intake [22, 29].

Testosterone has been shown to have a direct, positive effect on bone healing by inducing callus formation and improving the biomechanical stability of fractured bone [30, 31]. As testosterone is aromatized to estrogen, it not only acts on bone directly via ARs but also indirectly via estrogen receptors (ERs) [9, 32]. Previous reports showed that men with mutations in ERs or aromatase genes suffered from severe osteoporosis, indicating that aromatization of testosterone to estradiol is essential for skeleton homeostasis [32–34]. In contrast to T, enobosarm does not aromatize to estrogen and acts solely via the ARs on bone [9, 16]. This could explain the different effects of T compared with EN on bone healing in our study.

In the present study, we observed a positive effect of enobosarm on bone healing applied immediately after Orx (EN-pr) in male rats. Increased callus area formed during the first 3 weeks of healing and decreased cortical density compensated by enhanced callus density after 6 weeks of healing indicated an advantage of long-term EN treatment for bone healing. However, short-term EN treatment (EN-th) applied after the osteotomy affected bone healing negatively. In the ovariectomized rat model, EN applied at dosages of 0.4 and 4 mg/kg BW improved osteoporotic bone tissue [17], where the higher dosage (4 mg/kg BW) had a stronger effect on bone healing than the lower one (0.4 mg/kg BW) [18]. However, the latter showed fewer uterotrophic effects and was therefore chosen in this study to minimize possible side effects. In contrast to the present data, EN applied after osteotomy enhanced callus formation and did not impair bone healing in the ovariectomized rat model [18]. Furthermore, the weight of the gastrocnemius muscle and BW were significantly enhanced after EN treatments in the female rat model [18]. This is different from the present data showing the lack of EN effects on the weight of leg muscles and BW in male rats. There are limited data on gender differences in response to SARM treatments. Experimental and clinical studies on EN reported either combined data for both genders or single-sex data [9, 12, 13, 17, 18]. A study on another non-steroidal SARM, GSK2881078, demonstrated enhanced sensitivity of muscle tissue in women compared to men [35]. A favorable influence of EN on bone healing observed in female rats could be positively enhanced by its indirect effect on muscle tissue along with the direct effect via ARs [18]. Besides the gender effect, the effect of rat age could contribute to the differences in bone healing under EN treatments. Growing females used in the previous study (3 months old) [18] may have responded to the EN treatments differently from aged male rats used in this study.

In the present study, we found that levator ani weight was significantly elevated by both EN treatments, whereas the weight of the gastrocnemius and soleus muscles was not changed. The different response of levator ani muscle and other skeletal muscles was also previously reported after administration of testosterone in juvenile male rats [36]. The levator ani muscle of the rat is widely used as an index of myotrophic activity of steroidal hormones and various SARMs [9, 28, 37]; it provides a sensitive and rapid assessment of the anabolic activity of substances [9]. However, the use of levator ani muscle as a single indicator of the anabolic effect of substances is critical [9], and other skeletal muscles have to be considered.

In our study, the anabolic effect of EN on the levator ani muscle was stronger (EN-pr: 89% and EN-th: 84% of the Non-Orx group) than its androgenic effect on the prostate (EN-pr: 51% and EN-th: 32% of the Non-Orx rats). The weight of the prostate was significantly increased solely after long-term treatment with EN-pr; there was no significant change following either short-term treatment (EN-th) or both testosterone treatments. In accordance with our data, other preclinical studies on arylpropionamide SARMs, including andarine and ostarine (enobosarm), reported increased weight of the levator ani in Orx rats to the level of sham-operated animals and only partially increased the weight of the prostate. Furthermore, enobosarm was more potent than testosterone propionate at the levator ani in rats [9, 11, 38, 39], which is in agreement with our data. The lack of androgenic activity of T treatments in the prostate can be explained by the limited bioavailability of oral testosterone propionate. This application was chosen based on previous studies that showed the favorable effect of T on bone [19–21].

The serum level of OC, a marker for bone turnover, was significantly reduced after both EN treatments (pr and th) in the present study. Similarly, Gao et al. [40] reported decreased serum OC levels after treatments with SARM S4 (andarine) in the castrated male rat and suggested its antiresorptive activity. In-vitro studies showed that EN induced differentiation of bone marrow cells to osteoblasts and inhibited osteoclast differentiation [38]. Thus, we hypothesized that EN applied in Orx rats could also have an antiresorptive activity slowing bone turnover, which would be advantageous in high-turnover osteoporosis; however, it might be a disadvantage if applied directly after the fracture. Indeed, anti-osteoporotic drugs such as bisphosphonates, with their known effect of decreasing bone turnover, impair or slow bone healing and are not prescribed for patients with fractures [41, 42]. Further detailed histomorphological analysis of bone tissue could help to clarify the in-vivo effects of EN on bone cells, osteoblasts, and osteoclasts.

Enhancement of serum phosphorus levels to the level of Non-Orx healthy rats after long-term treatments with T and EN was recorded. Vanderschueren et al. [26] reported low serum phosphate levels and diminished bone parameters in Orx rats, whereas testosterone treatment restored these parameters. An elevated serum phosphorus level after enobosarm treatment was also measured in ovariectomized rats [18]. This systemic effect of EN should be clarified in further studies.

To summarize, EN-pr applied after Orx improved several parameters of bone healing. However, the overall impact of EN-pr treatment on bone healing was less than that of T. EN-pr showed an additional side effect on the prostate. EN-th applied after osteotomy appeared to have an unfavorable effect on bone healing. The weight of the levator ani muscle was increased after both EN treatments, indicating localized anabolic activity; however, other skeletal muscles did not respond to these treatments. T improved bone healing irrespective of the duration of the treatments (th and pr) and without having an effect on the prostate or the levator ani muscle.

The limitation of the present study is the investigation of fracture healing at one point of time. The time point was set at 6 weeks after osteotomy, enabling analysis of callus formation before callus resorption occurred and assessment of biomechanical properties of calcified callus [22].

In conclusion, the results of this study indicate that EN-pr treatment could be further investigated as a therapy for bone healing in men; its side effects should, however, be closely examined. Based on our data, administration of the SARM enobosarm immediately after fracture is not advisable in aged male rats. Furthermore, gender-specific differences in the response of the musculoskeletal system to SARMs or steroid hormones should be evaluated in future research. Testosterone applied as hormone replacement therapy has a positive effect on bone healing; however, its clinical application is limited due to its side effects [6].

## Electronic supplementary material

Below is the link to the electronic supplementary material.

Supplementary file1 (PPTX 454 kb)
