# Supplementary material for: Effect of Selective Androgen Receptor Modulator Enobosarm on Bone Healing in a Rat Model for Aged Male Osteoporosis
Source: Calcif Tissue Int. 2020 Sep 2;107(6):593–602. doi: 10.1007/s00223-020-00751-x (PMC7593387; doi:10.1007/s00223-020-00751-x)
Supplement: Supplementary file 1 — Supplementary file1 (PPTX 454 kb) [file 223_2020_751_MOESM1_ESM.pptx]

## Slide 1
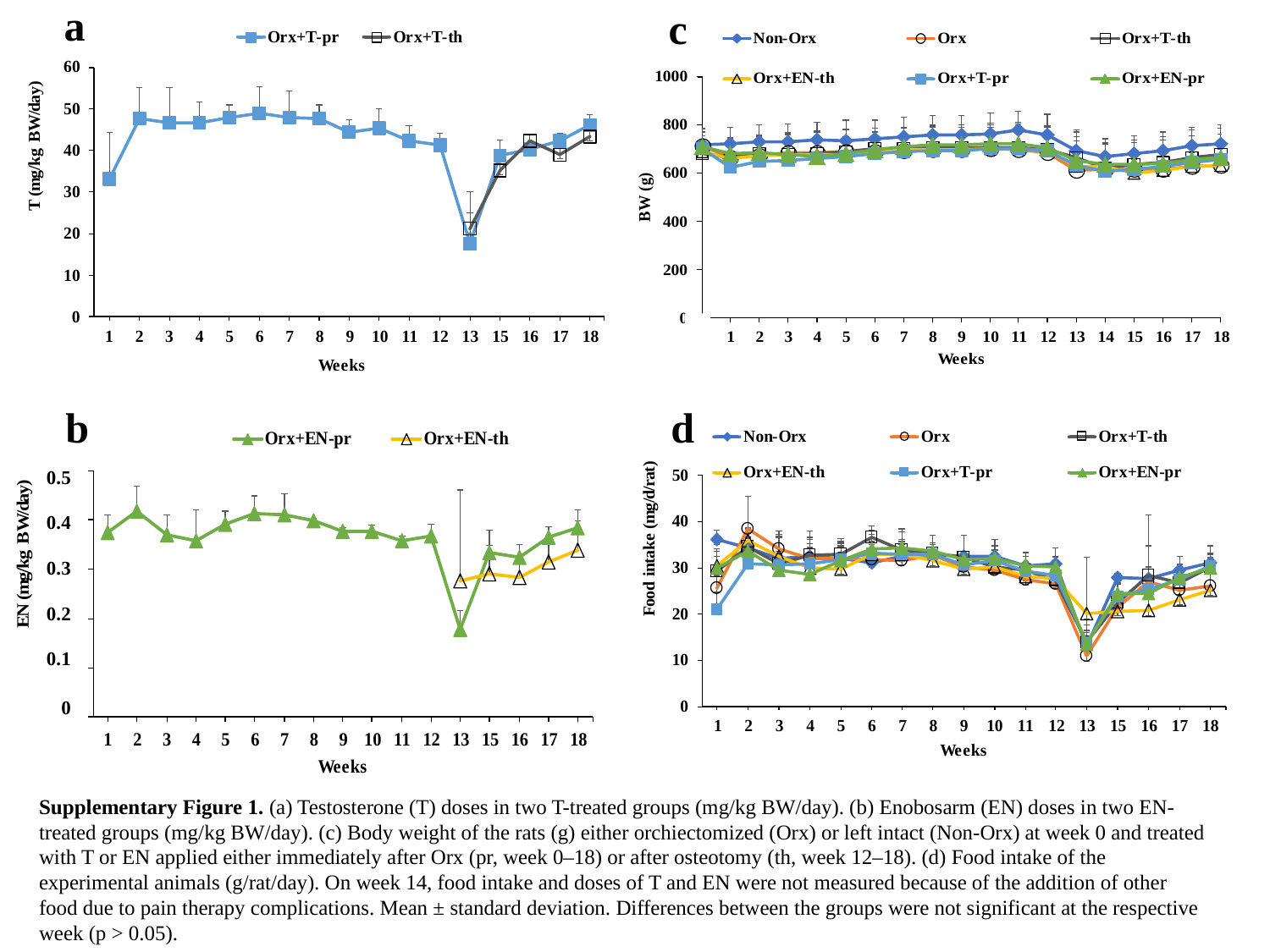

a
c
b
d
0.5
0.4
0.3
0.2
0.1
 0
Supplementary Figure 1. (a) Testosterone (T) doses in two T-treated groups (mg/kg BW/day). (b) Enobosarm (EN) doses in two EN-treated groups (mg/kg BW/day). (c) Body weight of the rats (g) either orchiectomized (Orx) or left intact (Non-Orx) at week 0 and treated with T or EN applied either immediately after Orx (pr, week 0–18) or after osteotomy (th, week 12–18). (d) Food intake of the experimental animals (g/rat/day). On week 14, food intake and doses of T and EN were not measured because of the addition of other food due to pain therapy complications. Mean ± standard deviation. Differences between the groups were not significant at the respective week (p > 0.05).
